# Temperature-Related Summer Mortality Under Multiple Climate, Population, and Adaptation Scenarios

**DOI:** 10.3390/ijerph16061026

**Published:** 2019-03-21

**Authors:** Jae Young Lee, Woo-Seop Lee, Kristie L. Ebi, Ho Kim

**Affiliations:** 1 Institute of Health and Environment and Graduate School of Public Health, Seoul National University, Seoul 08826, South Korea; jaeyoung.lee@alumni.stanford.edu; 2 Climate Services and Research Department, APEC Climate Center, Busan 48058, South Korea; wslee@apcc21.org; 3 Center for Health and the Global Environment, University of Washington, Seattle, WA 98105, USA; krisebi@uw.edu

**Keywords:** projection, future mortality, climate change, adaptation, population change

## Abstract

Projections of the magnitude and pattern of possible health risks from climate change should be based on multiple climate and development scenarios to describe the range of uncertainties, to inform effective and efficient policies. For a better understanding of climate change-related risks in seven metropolitan cities of South Korea, we estimated temperature-related summer (June to August) mortality until 2100 using projected changes in climate, population, and adaptation. In addition, we extracted the variations in the mortality estimates associated with uncertainties in climate, population, and adaptation scenarios using 25 climate models, two Representative Concentration Pathways (RCP 4.5 and 8.5), three population scenarios (high, medium and low variants), and four adaptation scenarios (absolute threshold shift, slope reduction in the temperature-mortality relationship, a combination of slope reduction and threshold shift, and a sigmoid function based on the historical trend). Compared to the baseline period (1991–2015), temperature-attributable mortality in South Korea during summer in the 2090s is projected to increase 5.1 times for RCP 4.5 and 12.9 times for RCP 8.5 due to climate and population changes. Estimated future mortality varies by up to +44%/−55%, −80%, −60%, and +12%/−11% associated with the choice of climate models, adaptation, climate, and population scenarios, respectively, compared to the mortality estimated for the median of the climate models, no adaptation, RCP 8.5, and medium population variant. Health system choices about adaptation are the most important determinants of future mortality after climate projections. The range of possible future mortality underscores the importance of flexible, iterative risk management.

## 1. Introduction

Projections of future temperature-related mortality associated with increasing surface temperatures under the Representative Concentration Pathways (RCPs) have infrequently considered future demographic change and the possible effectiveness of adaptation policies [1,2,3,4,5,6]. Using total population changes, Wu et al. (2014), Stone et al. (2014), Kim et al. (2014), and Martinez et al. (2016) projected future temperature-related mortality to be linearly scaled with population change [7,8,9,10]. Lee and Kim (2016) considered population and demographic composition changes, projecting that future temperature-related mortality rapidly increases in South Korea [11]. A few studies have started to consider adaptation in projections of future mortality. Gosling et al. (2017) reported future mortality changes for six major adaptation scenarios, showing the importance of incorporating estimates of the effectiveness of adaptation policies in temperature-related mortality projections [12]. Li et al. (2016) showed reductions in heat-related mortality when assuming both a slope reduction and minimum mortality temperature increase in the exposure–response relationships [13]. Petkova et al. (2017) extrapolated future adaptation trends based on historical temperature-mortality relationships when projecting future heat-related mortality [14].

Climate, demographic changes, and adaptation are major factors affecting future temperature-related mortality, and should be considered in projections to provide more precise estimates. We projected, for the first time, future summer temperature-related mortality (June to August) in seven major metropolitan cities of South Korea including climate, demographic changes, and adaptation. Metrics of adaptation were slope reduction of the exposure–response relationship, absolute threshold shift, and a combination of these two, based on Gosling et al. (2017) [12]. In addition, we analyzed historical mortality and temperature data in South Korea during 1991–2015 to quantify the rate of adaptation. We projected temperature-related mortality using 25 general circulation models (GCMs), two greenhouse gas emission pathways (RCP 4.5 and 8.5), three population scenarios (high, medium and low variants), and four adaptation scenarios, to describe the range of projected mortalities. Projected future mortality and its variations will be valuable in the assessment of climate change risks, and can inform policies to prepare for and manage risks.

## 2. Materials and Methods

### 2.1. Data Collection

We collected daily non-accidental mortality (ICD-10 codes A00-R99) and daily maximum temperature and humidity during 1991–2015 for seven metropolitan cities of South Korea (Seoul, Busan, Daegu, Incheon, Gwangju, Daejeon, and Ulsan, see Figure S1 for a map) from Statistics Korea and the Korea Meteorological Administration, respectively. Table S1 shows the descriptive statistics of city-specific summer temperature during 1991-2015. Projected daily maximum temperatures were obtained from the Asia-Pacific Economic Cooperation Climate Center in Busan, South Korea. Future temperatures were projected to 2100 using 25 GCMs under RCP 4.5 and 8.5. Table S2 summarizes the general information on the 25 GCMs used. RCP 4.5 is a mitigation scenario, while RCP 8.5 is a high-end emissions business-as-usual scenario. Figure S2 shows the projections of global CO_2_ concentrations based on the emissions scenarios described under the four RCPs. Data for demographic and population changes over the period 2000–2100 were obtained from the United Nations [15]. In population data, three (high, medium, and low) fertility variants were used. Under the high and low fertility variants, fertilities are projected to remain 0.5 children above and below the medium fertility, respectively. A detailed description of assumptions can be found in United Nations [15].

### 2.2. Temperature-Mortality Relationship

Exposure–response relationships for the seven metropolitan cities were quantified using a distributed lag nonlinear model assuming a quasi-Poisson distribution [16]. The model can be expressed as

log(E[Mortality]) = CB(T) + DOW + NS(time)

Here, CB(T) is the cross-basis matrix of the daily maximum temperature and lag based on natural cubic splines with five degrees of freedom, DOW is the day of the week, and NS(time) is the natural cubic spline function of time. The lag is modeled up to 21 days. This model was used in Lee and Kim’s study [11]. To account for the demographic change, the total population was divided into two age groups: (1) 0–69 years; and (2) ≥70 years. The age of 70 was chosen because it divided total non-accidental mortality roughly into half. Temperature-mortality curves were separately quantified for each age group.

### 2.3. Temperature-Related Mortality Projection in Summer

Future temperature-related mortality in summer (from June to August) was projected for every 10-year period up to the 2090s. Future mortality is reported in terms of the mortality ratio (MR) that can be calculated by dividing the estimated future average daily mortality by the baseline average daily mortality over 1991–2015 [11]. Mortality was projected for 25 GCMs, RCP 4.5 and 8.5, and three population variants (high, medium, and low fertility variants). We chose summer as the target period for the projections because much less is known about adaptation to cold than to hot temperatures [4,7,8,9,10,12,14,17,18].

### 2.4. Adaptation

Four adaptation scenarios were examined to determine their consequences for projected temperature-attributable mortality. First, we used reductions in slope (10%, 20%, and 30%) and shifts in the absolute threshold (1 °C, 2 °C, and 3 °C) of the exposure–response relationship. These are the most popular methods for incorporating adaptation into projections because they are relatively simple and straightforward to apply [12,19,20,21,22]. A combination method that applied the slope reduction and absolute threshold shift simultaneously was also used (all nine combinations).

Despite their popularity, a common limitation with these methods is that the adaptation rate does not rely on historical data. To overcome this, we additionally applied a method similar to the one introduced by Petkova et al. (2017) [14]. We projected and compared the temperature-mortality relationships during the first (1991–2000) and last (2006–2015) ten years in the baseline period to understand trends in adaptation. The results were used to explore whether adaptation in South Korea occurred in the form of a slope reduction, a threshold shift, or both. Finally, we assumed an amount of adaptation at 2100 and used a sigmoid function-based interpolation to project future adaptation curves between the baseline period and 2100.

## 3. Results

**Figure 1:**
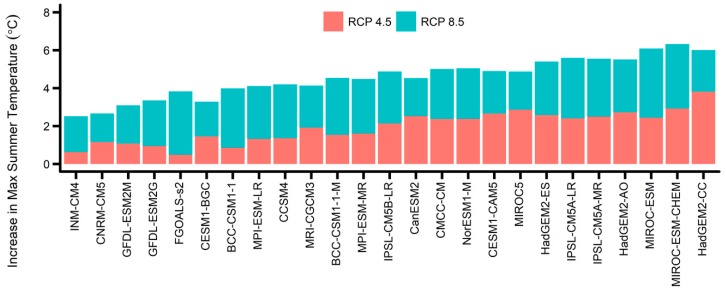
The average increase in maximum temperature under two Representative Concentration Pathways (RCPs) during summer time from the 2000s to the 2090s for each general circulation model (GCM), South Korea.

Figure 1 shows how much the 10-year average of maximum summer temperature increased from the 2000s to the 2090s for each GCM and RCP; the GCMs were sorted based on the average increase under the two RCPs. The smallest temperature increase was from the INM-CM4 model; the projected increase was 0.63 °C for RCP 4.5 and 2.51 °C for RCP 8.5. The largest temperature increase was from the HadGEM2-CC model; the projected increase was 3.81 °C for RCP 4.5 and 6.01 °C for RCP 8.5. Figures S3 and S4 show the average and 99-percentile daily maximum summer temperature for the 25 individual climate models, for each 10-year period from the 2000s to the 2090s under the two RCPs. The difference between the lowest and highest summer temperatures among the climate models in 2090s was 3.7 °C for RCP 4.5 and 4.2 °C for RCP 8.5. The city-specific temperature projections are shown in Figure S5; the temperature change trends were similar in all seven cities. The city-specific temperature increases from the 2010s to the 2090s averaged over the 25 GCMs under RCP 8.5 were 4.74, 4.70, 4.65, 4.56, 4.52, 4.42, and 4.29 °C for Seoul, Incheon, Daejeon, Daegu, Gwangju, Ulsan, and Busan, respectively.

Temperature-mortality relationships for each city (Figure S6) showed that mortality increased rapidly at extremely high temperatures, while it increased slowly or stayed roughly constant at low temperatures.

Further, the slope of the exposure–response curve at high temperatures above the minimum mortality temperature (31 °C) was much higher for the older age group compared to that for the younger age group, indicating that older adults were more vulnerable to hot temperatures than the younger population.

**Figure 2:**
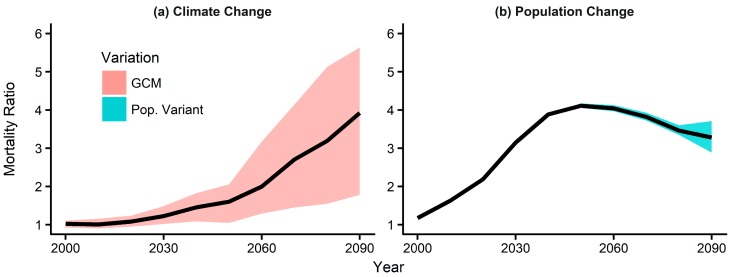
Projections of summer temperature-attributable mortality ratio (MR) in seven major cities of South Korea until the 2090s based on (**a**) climate under RCP 8.5 and (**b**) population changes. The red and blue shaded areas show the variations in the MR projections due to uncertainties in climate and population changes, respectively.

Based on these city-specific and age group-specific exposure–response relationships and projected daily maximum temperature, we calculated the MR in the summer for every 10-year period until the 2090s. Future MRs due to climate and population changes are shown separately in Figure 2. In Figure 2a, the median MR projected based on the 25 GCMs under RCP 8.5 gradually increases to around 3.9 in the 2090s. This trend is somewhat expected because the temperatures projected under the GCMs also gradually increase. The red shaded area represents the MR variations associated with the variations in temperature projected by the climate models. The upper and lower boundaries of the shaded area indicate the 95th and 5th percentiles of MR projections, respectively. On the other hand, the MR variations associated with population change peak around the 2050s and then decrease to about 3.3 in the 2090s (Figure 2b). The upper and lower boundaries of the shaded area indicate the high and low fertility variants. This trend is similar to the population prospect of the elderly age group (70+ years), which increases rapidly until 2060 and decreases thereafter (Figure S7). Figure S8 shows the breakdown of population-change-induced MR in Figure 2b by age groups. As can be seen, most of the mortality increase due to population change is driven by the increase of the elderly population. As shown in Figure 2, the variations due to the choice of climate model are much bigger than that due to population change (high, medium, and low fertility), and generally increase when projecting further into the future. This can be interpreted as that there is a greater uncertainty in the projections of future temperature than the projections of future populations, and that the further into the future, the larger the uncertainty. This shows it is very important to carefully consider which climate model(s) to use for mortality projections.

**Figure 3:**
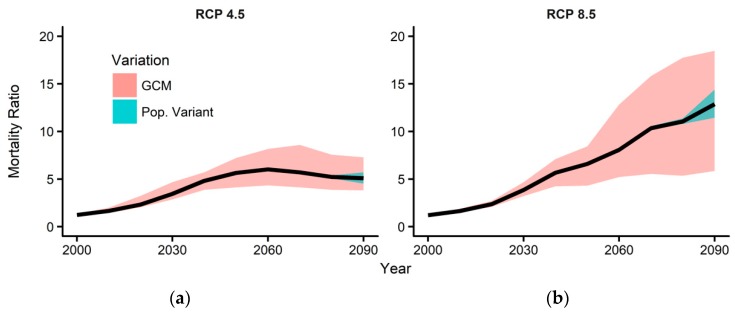
Projections of summer temperature-attributable mortality ratio (MR) associated with climate and population changes from 2000 to 2100 under (**a**) RCP 4.5 and (**b**) 8.5. The red and blue shaded areas show the variations in the MR projections due to uncertainties in climate and population changes, respectively.

Figure 3 shows the overall MR accounting simultaneously for climate and population changes. The solid black line is the median projection value among the GCMs under the medium population variation. The red and blue shaded areas represent the MR variations associated with the climate models and population change scenarios, respectively, as in Figure 2. Under RCP 4.5, the MR peaks in or around the 2060s and slightly declines to 5.1 in the 2090s because of the projected population decrease. Under RCP 8.5, MR increases to 12.9 in the 2090s because of the increase in MR associated with climate change cancels the decrease in MR associated with population change during the late 21st century. The variation associated with the choice of climate model was much bigger than that associated with population scenarios. Further, the GCM associated variations were much bigger for RCP 8.5 than that for RCP 4.5. During the 2090s, the range of MR varied from 3.8 to 7.3 associated with variations in climate models under RCP 4.5, and varied from 5.9 to 18.5 under RCP 8.5.

**Figure 4:**
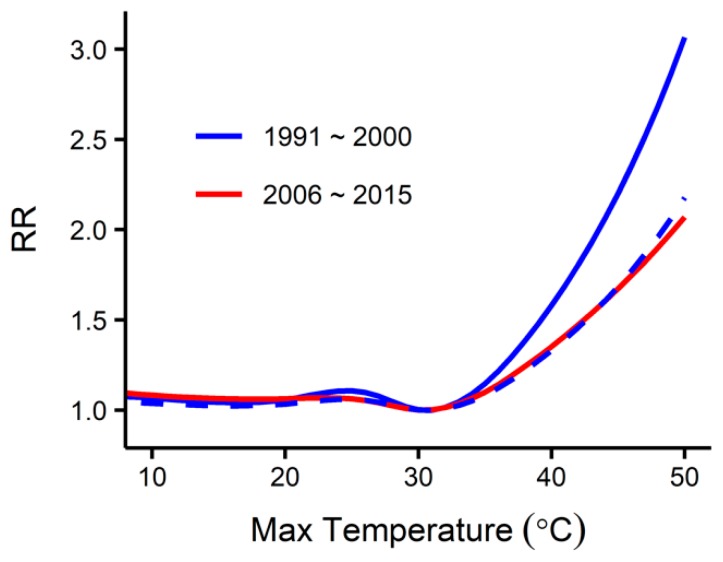
The overall temperature-mortality relationships during the first (1991–2000) and the last (2006–2015) ten years of the baseline period created using DLNM. The x-axis represents the daily maximum temperature, and the y-axis represents the overall cumulative effects of the maximum temperature on mortality. The dashed curve shows the 43% slope reduced version of the relationship during 1991–2000.

**Figure 5:**
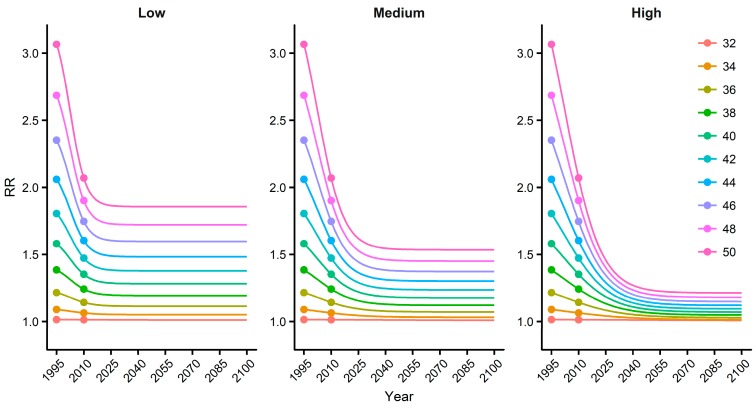
Temperature-specific adaptation curves during 1995–2100. The points in 1995 and 2010 represent the relative risks (RR) for each temperature during the periods 1991–2000 and 2006–2015, respectively.

We estimated how adaptation could alter future temperature-related mortality by first analyzing historical data to understand how people adapted to temperature in South Korea. Figure 4 shows the historical temperature-mortality relationship during the first (1991–2000) and the last (2006–2015) ten years of the baseline period. The relative risk above the minimum mortality temperature (31 °C) in the last ten years is approximately 43% smaller than that in the first ten years. Such rapid adaptation may be due to the wide introduction of air conditioning, medical advancements, and improvements in housing. It is interesting to note that while the slope of the relationship declined, the minimum mortality temperature remained constant. To project future adaptation based on the historical data, we assumed three adaptation scenarios for the 2090s: 80% (high adaptation), 50% (medium adaptation), and 20% (low adaptation). The extent of adaptation between the baseline period and the 2090s was defined by a sigmoid function [14]. Figure 5 shows the resulting temperature-specific adaptation curves during 1995–2100.

**Figure 6:**
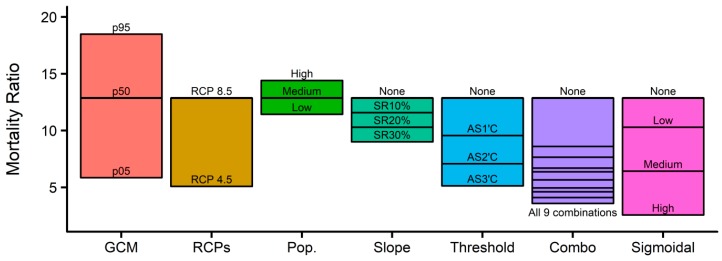
Variations in mortality ratio associated with multiple climate, population, and adaptation scenarios in the 2090s (2090–2099). Orange, dark yellow, green, turquoise, light blue, purple, and magenta colors represent the range of variation associated with variations in GCMs, RCPs, population, and four adaptation scenarios (slope reduction, absolute threshold shift, the combination of the slope reduction and the threshold shift, and the sigmoid function), respectively.

Figure 6 visualizes how climate, population, and adaptation scenarios change the projections of future summer mortality in the 2090s. Adaptation is expected to significantly reduce future temperature-related mortality. Mortality is more sensitive to a shift in the absolute threshold than to the slope reduction; the impact of a 1 °C shift is larger than that of a 20% reduction in the slope. The four adaptation scenarios (slope reduction, the absolute threshold shift, the combination, and sigmoidal function) can reduce the reference MR of 12.9 (median GCM, RCP 8.5, medium population, and no adaptation) by up to 1.4, 2.5, 3.6, and 5.0 times, respectively. As shown in Figure 6, source-specific variations can be compared with each other. In the 2090s, the MR variation associated with climate models is the largest (+44%/−55%), followed by that associated with adaptation (−80%), climate (−60%), and population scenarios (+12%/−11%) compared to the reference MR of 12.9. The MR variation trend in the 2050s is similar to that in the 2090s, as shown in Figure S9.

## 4. Discussion

This study projected future temperature-related mortality in seven major cities of South Korea, considering climate, demographic change, and adaptation. In addition, we comprehensively assessed and compared variations in future mortality estimates associated with uncertainties in the climate model, climate, population, and adaptation projections. The most important source of future variation in temperature-related mortality is associated with variations in projected climate change, followed by the uncertainties in adaptation, while the mortality variation associated with uncertainties in population change is relatively small.

The uncertainty in climate change largely stems from the variations among climate models: the projected average daily maximum summer temperature in the 2090s varies up to 3.7 °C for RCP 4.5 and 4.2 °C for RCP 8.5 across 25 GCMs. Therefore, the choice of climate model is critical in assessing the risk of climate change. Because selecting the most informative climate model is a difficult task, many studies use multiple climate models and use the median value projected using those models. Li et al. (2016) [13] used 31 climate models and Petkova et al. (2013, 2017) [2,14] used 33 climate models. However, the majority of projections relied on a single model, which may under- or over-estimate the median value from a range of climate models.

The uncertainty in population projections is relatively small. Population can be defined by three components: fertility, mortality, and migration. The relationships between the population and these components are relatively simple and there are smaller uncertainties as to how fertility, mortality, and migration will change in the future in South Korea.

Lastly, the uncertainty in future mortality associated with the various adaptation scenarios is large because little is known about how fast and how much people can adapt to future climates. Most previous projections relied on arbitrarily selected adaptation scenarios or scenarios based on historical trends that may or may not continue. Because of the uncertainties in adaptation scenarios, it is worth examining a range of possibilities, to inform adaptation planning with an iterative risk management framework.

## 5. Conclusions

To precisely assess the health risks of climate change, not only climate change but also population changes and the amount of adaptation must be projected. This study is the first to include all three factors in projecting future temperature-attributable mortality and its variation in seven cities of South Korea. The mortality ratio increases to 5.1 for RCP 4.5 and 12.9 for RCP 8.5 in the 2090s in South Korea associated with demographic and climate change. The MR variations associated with climate models, adaptation, climate, and population scenarios are +44%/−55%, −80%, −60%, and +12%/−11%, respectively. These results highlight the importance of climate change and adaptation scenarios, and encourage further efforts to quantify the sources of uncertainties in projections, to inform risk management approaches.

## Supplementary Materials

The following are available online at https://www.mdpi.com/1660-4601/16/6/1026/s1, Figure S1: Map of South Korea and the locations of the seven cities, Figure S2: Projection of global CO_2_ concentrations under four RCP scenarios. Black line indicates the historical global CO_2_ concentrations (1960–2018). The data is obtained from http://data.okfn.org/data/core/co2-ppm and http://www.iiasa.ac.at/web-apps/tnt/RcpDb, Figure S3: The 10-year-average daily maximum summer (Jun–Aug) temperature in South Korea under RCP 4.5 and 8.5 from 2000s to 2090s, Figure S4: The 99-percentile daily maximum summer (Jun–Aug) temperature in South Korea under RCP 4.5 and 8.5 for each 10-year period from 2000s to 2090s, Figure S5: The city-specific future summer temperature changes from 2000s to 2090s, Figure S6: The city-specific relationship between daily maximum temperature and mortality for each age group, Figure S7: The population prospects of two age groups (0 to 69 years and 70+ years) in South Korea by United Nations (UN) until 2100 under three fertility variant scenarios (high, medium, and low), Figure S8: The breakdown of population-change-induced mortality ratio by age groups (0 to 69 years and 70+ years) in South Korea until 2100 under three fertility variant scenarios (high, medium, and low), Figure S9: Variations in mortality ratio stemming from multiple climate, population and adaptation scenarios in the 2050s (2050–2059). Orange, dark yellow, green, turquoise, light blue, purple and magenta colors represent the range of variation due to the variations in GCMs, RCPs, population, and four adaptation scenarios (slope reduction, absolute threshold shift, the combination of the slope reduction and the threshold shift and the sigmoid function), respectively, Table S1: The city-specific minimum, average, and maximum summer temperature during the baseline period (1991–2015), Table S2: GCMs used in this study. This table contains model name, modelling center that developed each model and country information.
