# Supplementary material for: Temperature-Related Summer Mortality Under Multiple Climate, Population, and Adaptation Scenarios
Source: Int J Environ Res Public Health. 2019 Mar 21;16(6):1026. doi: 10.3390/ijerph16061026 (PMC6466250; doi:10.3390/ijerph16061026)
Supplement: Supplementary file 1 [file ijerph-16-01026-s001.pdf]

International Journal of
Environmental Research and Public Health

MDPI

Temperature-related summer mortality under multiple climate, population and adaptation scenarios

Jae Young Lee, Woo-Seop Lee, Kristie L. Ebi and Ho Kim

Table S1. The city-specific minimum, average, and maximum summer temperature during the baseline period (1991–2015).

City	Minimum (°C)	Average (°C)	Maximum (°C)
Seoul	10.8	24.6	38.4
Busan	12.4	23.7	35.8
Daegu	9.8	25.3	39.4
Incheon	11.9	23.6	37.2
Gwangju	8.7	24.9	38.5
Daejeon	8.1	24.6	37.7
Ulsan	10.8	24.3	38.8

Table S2. GCMs used in this study. This table contains model name, modelling center that developed each model and country information.

Model Name	Modelling Center	Country
BCC-CSM1.1	Beijing Climate Center Climate System Model	China
BCC-CSM1.1-M		
CanESM2	Canadian Centre for Climate Modelling and Analysis	Canada
CCSM4	National Center for Atmospheric Research	USA
CESM1-BGC	Community Earth System Model Contributors	USA
CESM1-CAM5		
CMCC-CM	Centro Euro-Mediterraneo sui Cambiamenti Climatici	Italy
CNRM-CM5	Centre National de Recherches Météorologiques	France
FGOALS-s2		
GFDL-ESM2G	NOAA Geophysical Fluid Dynamics Laboratory	USA
GFDL-ESM2M		
HadGEM2-AO	National Institute of Meteorological Research	S. Korea
HadGEM2-CC	Met Office Hadley Centre for Climate Science and Service	UK
HadGEM2-ES		
INM-CM4	Institute for Numerical Mathematics	Russia
IPSL-CM5A-LR	Institut Pierre-Simon Laplace	France
IPSL-CM5A-MR		
IPSL-CM5B-LR		
MIROC-ESM	Japan Agency for Marine-Earth Science and Technology, Atmosphere and Ocean Research Institute	Japan
MIROC-ESM-CHEM		
MIROC5	Atmosphere and Ocean Research Institute	Japan
MPI-ESM-LR	Max Planck Institute for Meteorology	Germany
MPI-ESM-MR		
MRI-CGCM3	Meteorological Research Institute	Japan
NorESM1-M	Norwegian Climate Centre	Norway

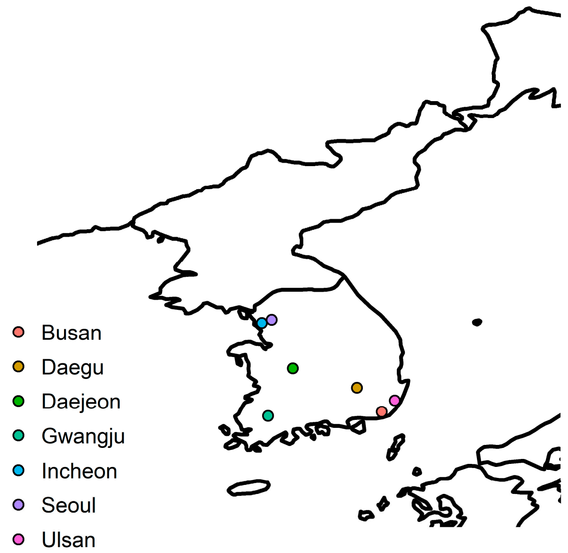

Figure S1. Map of South Korea and the locations of the 7 cities.

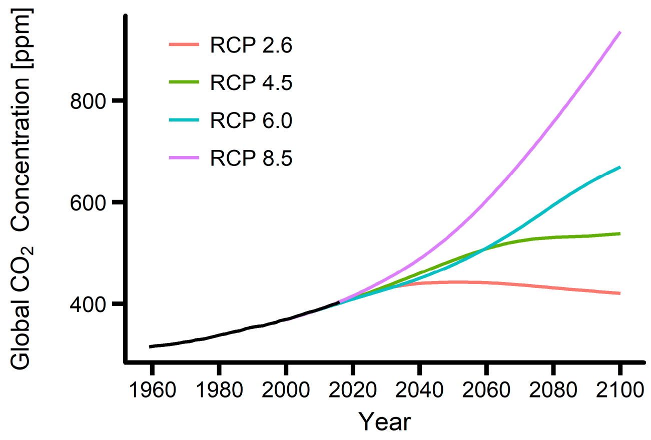

Figure S2. Projection of global CO_2 concentrations under four RCP scenarios. Black line indicates the historical global CO_2 concentrations (1960–2018). The data is obtained from http://data.okfn.org/data/core/co2-ppm and http://www.iiasa.ac.at/web-apps/tnt/RcpDb.

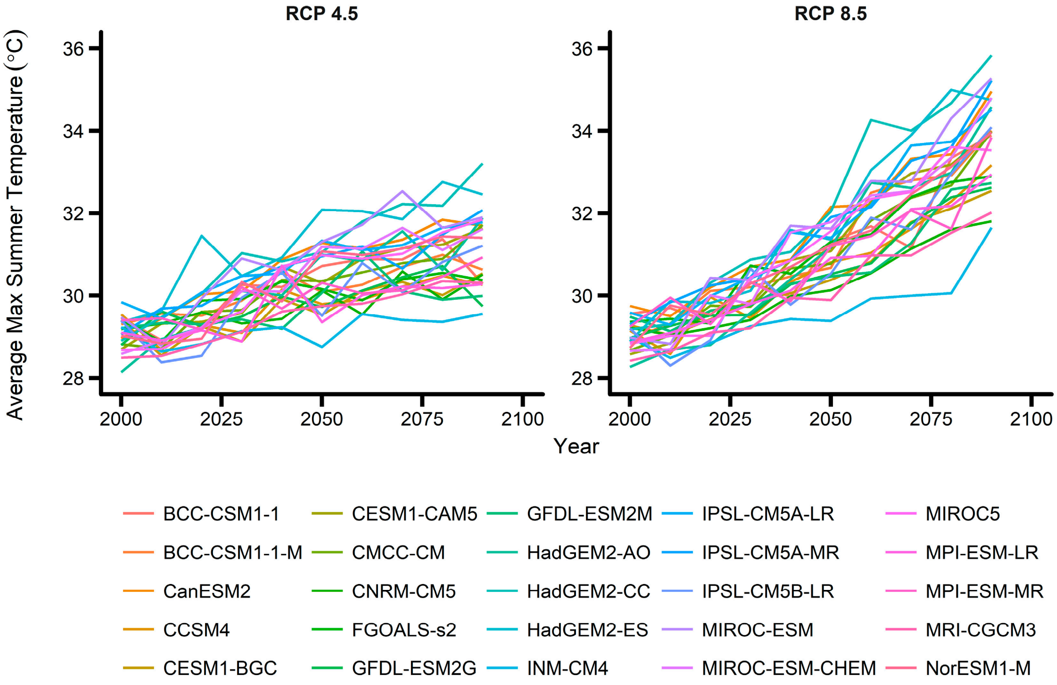

Figure 3. The 10-year-average daily maximum summer (Jun–Aug) temperature in South Korea under RCP 4.5 and 8.5 from 2000s to 2090s.

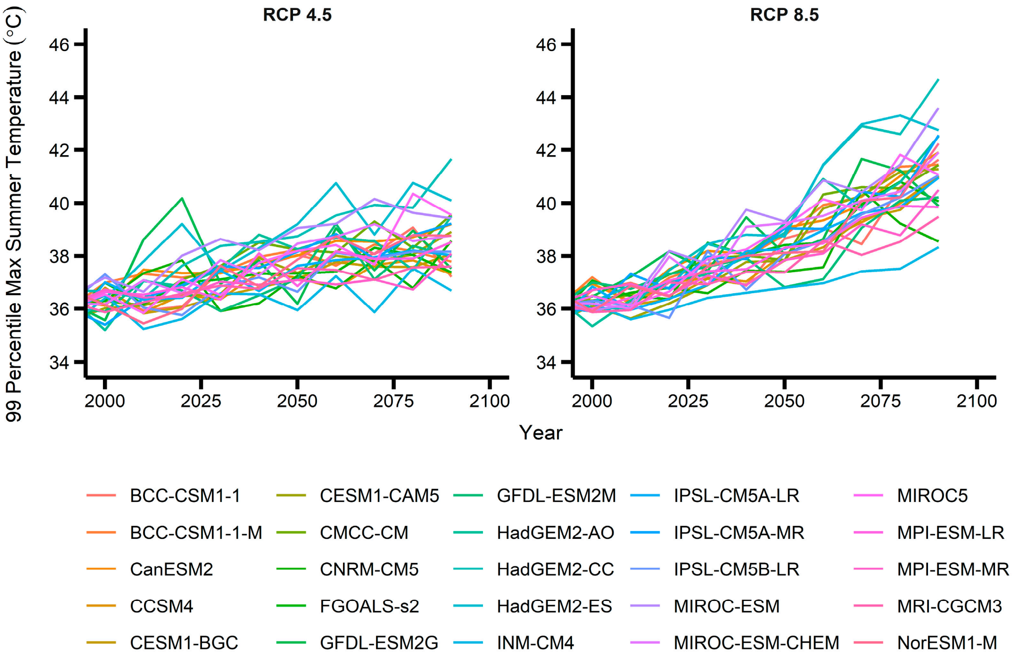

Figure S4. The 99-percentile daily maximum summer (Jun–Aug) temperature in South Korea under RCP 4.5 and 8.5 for each 10-year period from 2000s to 2090s.

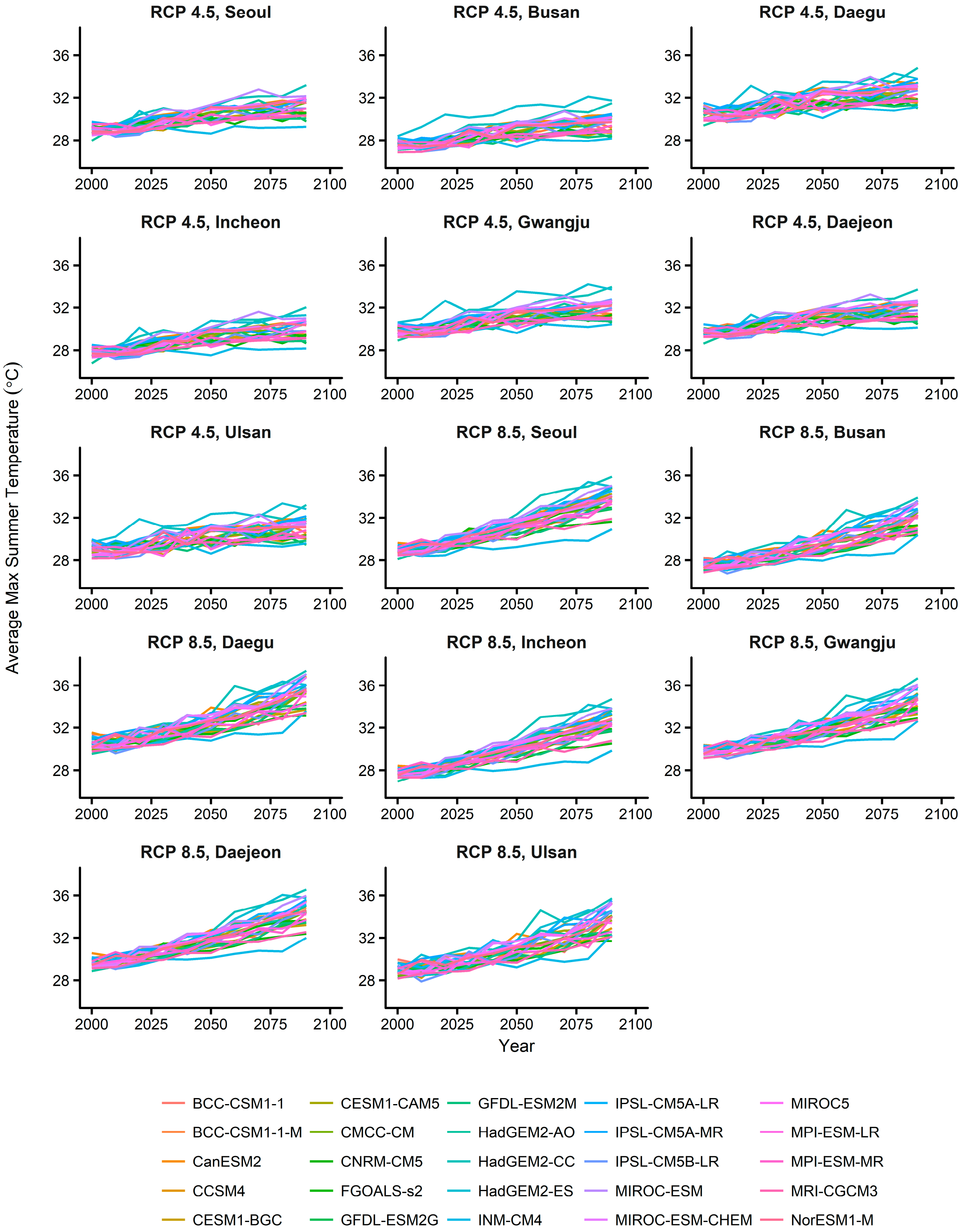

Figure S5. The city-specific future summer temperature changes from 2000s to 2090s.

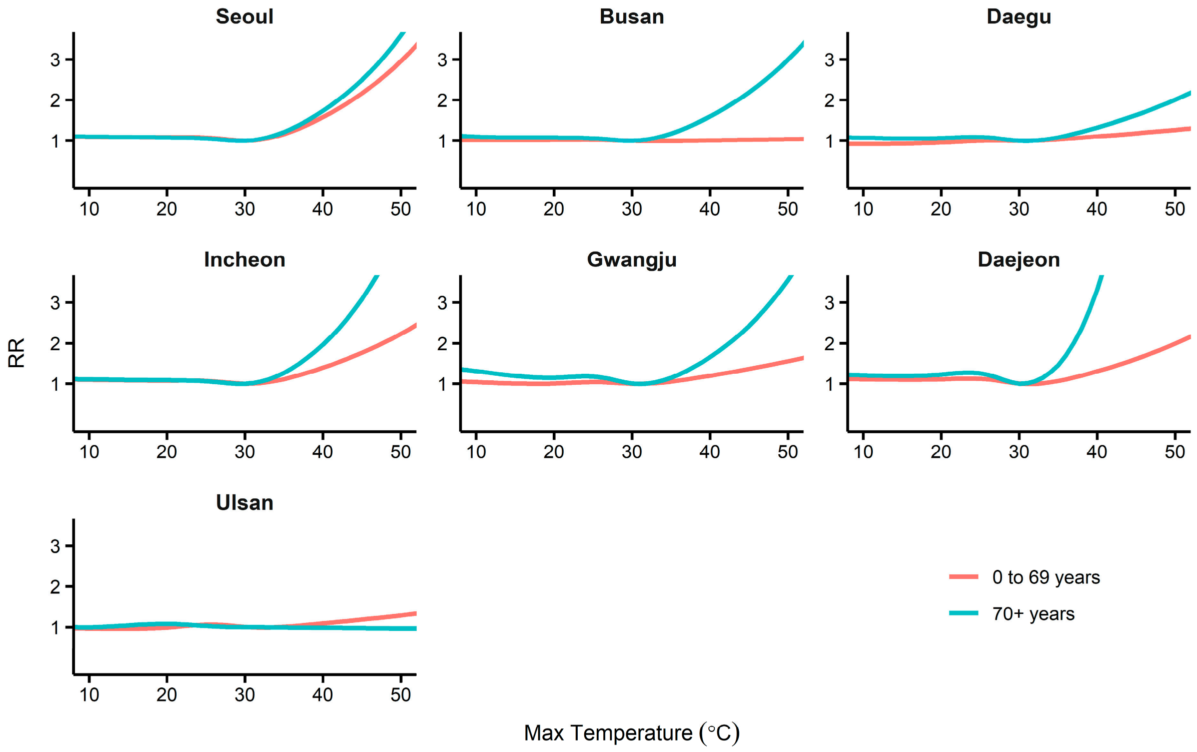

Figure S6. The city-specific relationship between daily maximum temperature and mortality for each age group.

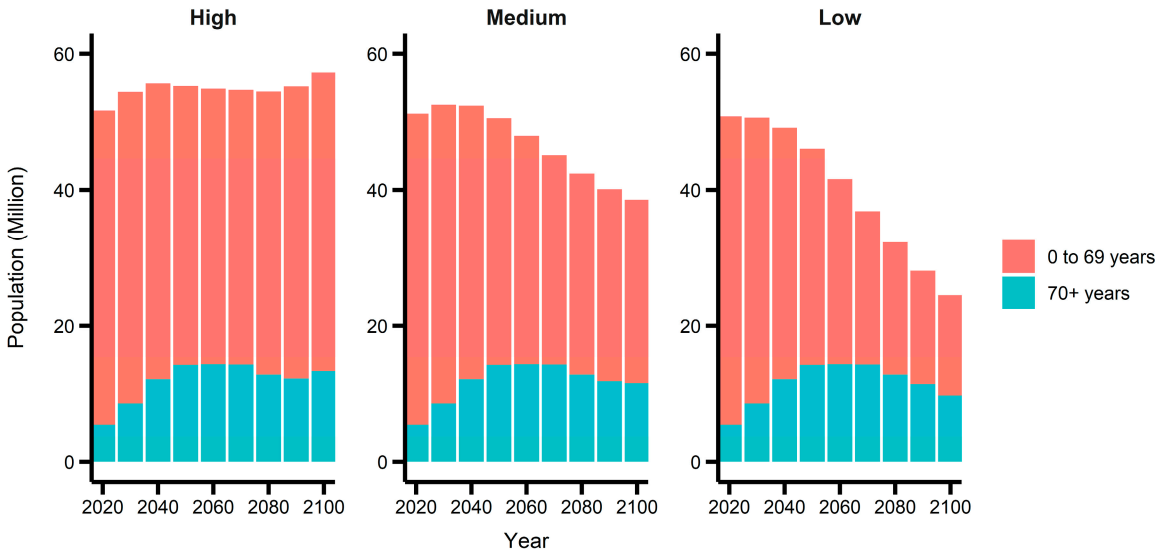

Figure S7. The population prospects of two age groups (0 to 69 years and 70+ years) in South Korea by United Nations (UN) until 2100 under three fertility variant scenarios (high, medium, and low).

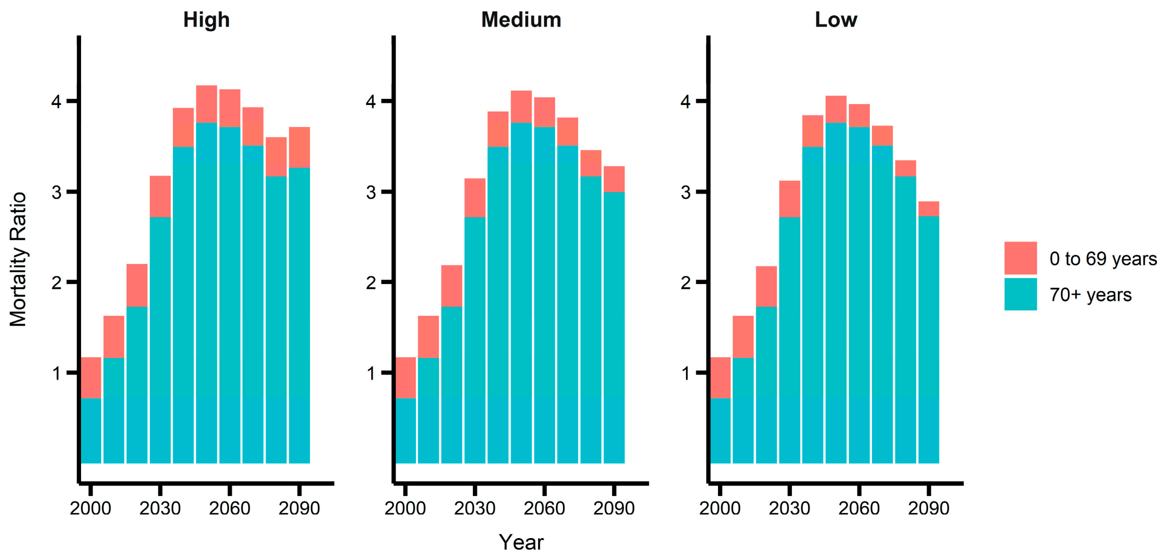

Figure S8. The breakdown of population-change-induced mortality ratio by age groups (0 to 69 years and 70+ years) in South Korea until 2100 under three fertility variant scenarios (high, medium, and low).

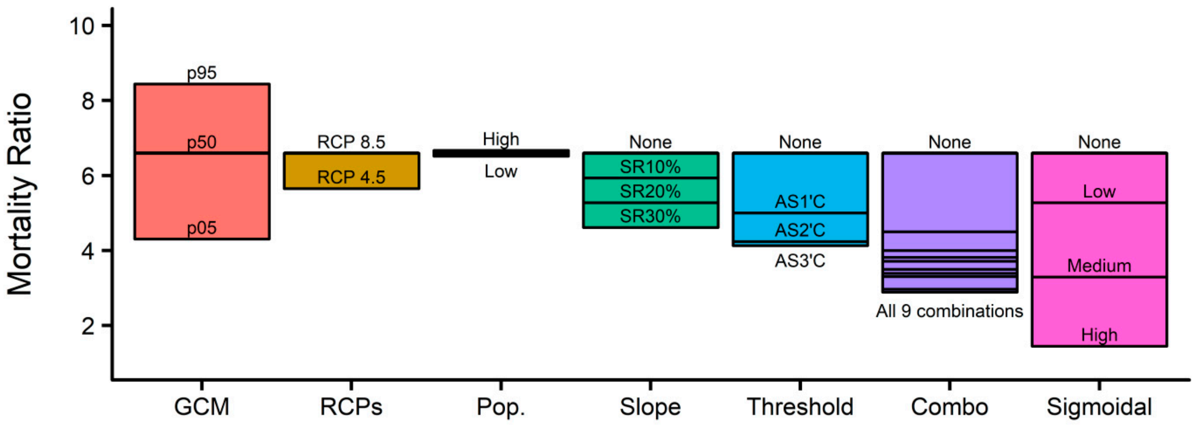

Figure S9. Variations in mortality ratio stemming from multiple climate, population and adaptation scenarios in the 2050s (2050–2059). Orange, dark yellow, green, turquoise, light blue, purple and magenta colors represent the range of variation due to the variations in GCMs, RCPs, population, and four adaptation scenarios (slope reduction, absolute threshold shift, the combination of the slope reduction and the threshold shift and the sigmoid function), respectively.
